# Enhancement of Efficiency of a TiO_2_-BiFeO_3_ Dye-Synthesized Solar Cell through Magnetization

**DOI:** 10.3390/ma15186367

**Published:** 2022-09-13

**Authors:** Hyun Sik Kang, Woo Seoung Kim, Yuwaraj K. Kshetri, Hak Soo Kim, Hak Hee Kim

**Affiliations:** 1 Department of Environmental and Bio-Chemical Engineering, Sun Moon University, Asan 31460, Chungnam, Korea; 2 Research Center for Eco Multi-Functional Nano Materials, Sun Moon University, Asan 31460, Chungnam, Korea

**Keywords:** BiFeO_3_, TiO_2_, magnetization, Hall effect, dye-sensitized solar cell

## Abstract

Enhancement in the efficiency of a TiO_2_ dye-sensitized solar cell (DSSC) has been demonstrated by introducing ferromagnetic perovskite BiFeO_3_ and controlling the magnetic field, which induces two-dimensional material-like properties in the bulk of the TiO_2_-BiFeO_3_ DSSC (a 3-dimensional material). The effect of the concentration of BiFeO_3_ as well as the magnetization direction on the performance of the TiO_2_-BiFeO_3_ DSSC has been investigated. After magnetization, it was confirmed that the current density, efficiency, and open circuit voltage of the TiO_2_-BiFeO_3_ DSSC were increased. The observed phenomena have been explained in terms of the Hall effect which is responsible for the reduction of the degree of freedom of the electron movement resulting in the two-dimensional material-like properties in the bulk of the TiO_2_-BiFeO_3_ DSSC.

## 1. Introduction

Currently, research on the commercialization of various two-dimensional materials is in progress [1]. Two-dimensional materials can be applied to various industrial fields such as the Internet of Things, curved devices, ultra-low power devices, next-generation batteries, water filters, and spacecraft [2,3,4]. As production costs fall sharply due to continuous research and development, the commercialization of two-dimensional materials is expected to be possible soon. However, these two-dimensional materials are mostly manufactured by mechanical peeling, liquid phase exfoliation, chemical vapor deposition, etc. These methods have disadvantages in that the production process is complicated and the yield is low due to the poor peeling efficiency of the layered material [5]. Therefore, it is difficult to use two-dimensional materials to fabricate an electronic device [6].

Nonetheless, two-dimensional materials have been extensively used in various fields such as displays, photocatalysis, hydrogen evolution, solar cells, semiconductors, etc [1]. There are many limitations of using two-dimensional materials as semiconductor components. In order to solve the limitations of two-dimensional materials, we propose a novel way of realizing two-dimensional-like material properties in three-dimensional bulk materials by combining a ferromagnetic material with the bulk semiconductor and then controlling the degree of freedom of the electron through magnetization [7,8,9,10,11].

**Scheme 1:**
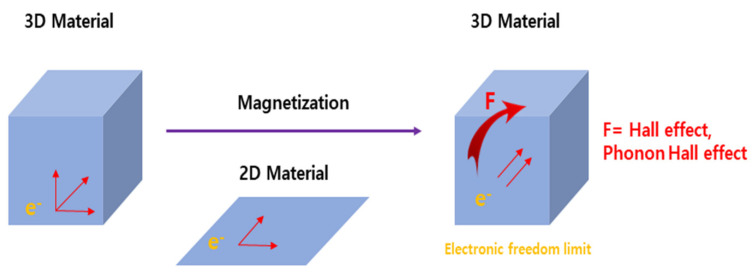
Schematic representation of 3D material with 2D material-like properties.

**Scheme 2:**
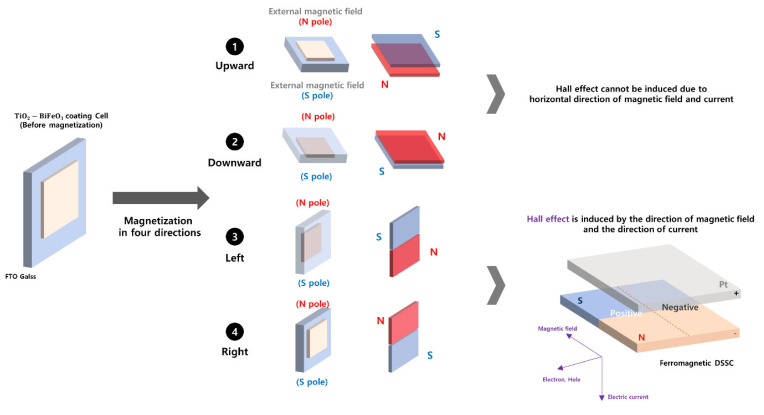
Schematic representation of the DSSC Hall effect according to the direction of the magnetic field.

The main objective is to introduce ferromagnetic perovskite BiFeO_3_ into a TiO_2_ semiconductor DSSC and to control the magnetic field so that the movement of electrons can be confined in a way similar to that in a two-dimensional material through the Hall effect. This technique reduces the degree of freedom of electrons and makes them move in two-dimensions. This movement ultimately reduces the scattering of electrons and the efficiency of the DSSC can be improved. Experimental apparatus has been designed to control the degree of freedom of electrons by magnetizing a three-dimensional material mixed with ferromagnetic materials, as shown in Scheme 1. When the sample is placed in the magnetic field, an electric field or potential difference occurs in a direction perpendicular to both the magnetic field and the current when the current flows in the direction perpendicular to the magnetic field as a result of the Hall effect [12]. By controlling the direction of the current and the magnetic field in the ferromagnetic DSSC, the polarization caused by the Hall effect is induced to control the degree of electron freedom as schematically represented in Scheme 2.

As a proof of concept of the above principle, we report in this paper a TiO_2_-BiFeO_3_ DSSC with enhanced efficiency by introducing two-dimensional-like material properties by controlling magnetic field. We first mix ferromagnetic perovskite BiFeO_3_ with the TiO_2_ semiconductor and fabricate the DSSC from the TiO_2_-BiFeO_3_ composite [13,14,15]. The magnetic field is then used to control the electron movement in the DSSC through the Hall effect producing two-dimensional-like material properties in the bulk of a TiO_2_-BiFeO_3_ DSSC. We confirm the possibility of manufacturing 3D materials with low electron degrees of freedom as in 2D materials through the control of electron mobility using a magnetic field. In addition, by selecting a perovskite BiFeO_3_ semiconductor material having a high Curie temperature [16] and maintaining ferromagnetism at room temperature, the DSSC is designed to be utilized at room temperature.

## 2. Materials and Methods

### 2.1. Synthesis of BiFeO_3_

The reagents 0.01 mol of Bi(NO_3_)_3_·5H_2_O, 0.01 mol of Fe(NO_3_)_3_·9H_2_O, and 0.02 mol (molar rate 1:1:2) of C_4_H_6_O_6_ were measured and mixed. Then 10% HNO_3_ solution was added to the mixture and subjected to ultrasonication and stirring in order to dissolve the mixture completely. The dissolved mixture was then dried at 45 °C using an evaporator. The powdered sample was ground into fine particles using an agate mortar. Each of the samples was calcined at 500 °C for 1 h, 500 °C for 6 h, and 500 °C for 6 h + 600 °C for 1 h using a muffle furnace. The heating rate was 5 °C/min. Three different BiFeO_3_ pastes with compositions of 1%, 3%, and 5% were prepared for the production of the ferromagnetic DSSC. The detailed experimental method of this experiment is shown in Figure S1 (Supplementary Information).

### 2.2. Synthesis of TiO_2_-BiFeO_3_ Paste

TiO_2_ (P-25), as-synthesized BiFeO_3_, ethyl cellulose (100 cP, 5), and α-terpineol (90%, Sigma Aldrich) were used to prepare TiO_2_-BiFeO_3_ paste. BiFeO_3_ 0.0032 mol and TiO_2_ 0.3168 mol were mixed and homogeneously ground using an agate mortar. The TiO_2_-BiFeO_3_ mixture was transferred to a beaker and 20 mL of ethanol was added. As the magnetic stirrer causes the BiFeO_3_ to magnetize and aggregate, non-magnetic stirring was carried out. Again, 3.33 g α-terpineol was added and stirred for 3 min, and 5 g of 10% ethyl cellulose solution was added. Finally, the solvent was removed by heating at 60 °C for 90 min using a vacuum evaporator to prepare the TiO_2_-BiFeO_3_ paste. Details of the synthesis process are shown in Figure S2 (Supplementary Information).

### 2.3. Fabrication of the TiO_2_-BiFeO_3_ DSSC

Fluorine-doped tin oxide (FTO) glass, TiO_2_ paste (Ti-Nanoxide T/SP, Solaronix, Aubonne, Switzerland), Iodolyte (AN-50, Solaronix) Pt paste (plastisol T/SP, Solaronix), Surlyn(thermoplastic hot-melt ionomer film), cover glass, and silver paste (60%, TED PELLA, Redding, CA, USA) were used for the DSSC fabrication. The FTO glass was used as a substrate because it exhibits good visible transparency owing to its wide band gap while retaining a low electrical resistivity due to the high carrier concentration caused by the oxygen vacancies and the substitutional fluorine dopant. First, the FTO glass was washed with water, ethanol, and acetone for 20 min. The TiO_2_-BiFeO_3_ paste was coated on a 0.5 cm × 0.5 cm area using the doctor blade. For this, a 65 µm thick scotch tape was used as a mask to create the 0.5 cm × 0.5 cm area. Therefore, the thickness of the coating was estimated to be 65 µm. The coated cell was calcined at 500 °C for 1 h. A dye solution was prepared using 0.03 g (0.025 moles) N719-Dye and ethanol 100 mL (stirring for 24 h). The sintered TiO_2_ cells were immersed in the prepared dyes for 24 h. While putting the Pt cell into the dye, the PTO cell is drilled with a size slightly larger than the size of the coated surface to manufacture the Pt cell. After washing in the same manner and coating with the plastisol solution, it is subjected to heat treatment at 400 °C for 30 min to produce a Pt cell. An appropriate size of Surlyn is cut and the DSSC is fabricated by assembling TiO_2_ and the Pt Cell. The cell is then heated at 100 °C for 5 min. Iodolyte (AN-50, Solaronix) with a concentration of 50 mM was used as an electrolyte. The electrolyte is injected into the manufactured DSSC cell, and the holes are closed with Surlyn and cover glass. A detailed manufacturing method is shown in Figure S3 (Supplementary Information).

### 2.4. Fabrication of the Magnetized TiO_2_-BiFeO_3_ DSSC

FTO glass, prefabricated TiO_2_-BiFeO_3_ paste, Iodolyte (AN-50, Solaronix), Pt paste (plastisol T/SP, Solaronix), Surlyn (thermoplastic hot-melt ionomer film), cover for the BiFeO_3_ DSSC glass, and silver paste (60%, TED PELLA) were used for manufacturing the DSSC [17]. FTO glass was washed for 20 min in the order of water, ethanol, and acetone to produce a conductive cell. On top of that, TiO_2_-BiFeO_3_ paste was applied on 0.5 cm × 0.5 cm size by doctor blade coating [18]. The coated TiO_2_-BiFeO_3_ cell was calcined at 500 °C for 1 h, and a dye solution was prepared using N719-Dye 0.03 g and 100 mL of ethanol (stirring for 24 h). The TiO_2_-BiFeO_3_ cell is magnetized according to the direction, as shown in Scheme 2. The magnetized cells are immersed in the prepared dyes for 24 h. While putting the Pt cell in the dye, the PTO cell was drilled with a size slightly larger than the size of the coated surface to manufacture the Pt cell. Then it was washed in the same manner and coated with plastisol solution to proceed to the firing at 400 °C for 30 min to produce a Pt Cell. To fix the TiO_2_-BiFeO_3_ cell and Pt Cell, an appropriate size Surlyn is cut and then assembled and heated (100 °C, 5 min). The electrolyte was injected into the manufactured DSSC cell, and the holes were closed with Surlyn and cover glass. A detailed manufacturing method is shown in Figure S4 (Supplementary Information).

### 2.5. Characterization

X-ray diffraction (XRD) spectra (Ultima IV, Rigaku, Tokyo, Japan) of the prepared sample were recorded with Cu Ka (λ = 1.5418 Å) radiation at room temperature to characterize its phase. Bragg angle 2θ was varied from 10° to 90°. UV-visible absorption and diffuse reflectance spectra (DRS) of the samples were measured using a UV–Visible spectrophotometer (Mecasys Optizen Alpha Smart Spectrometer, South Korea). Additionally, a solar simulator was used to measure DSSC efficiency and current density. The magnetization degree along the direction was measured with a vibrating sample magnetometer (VSM 7404). The morphology and particle size of the BiFeO_3_ ceramic were observed by field emission scanning electron microscopy (FE-SEM) (JSM-6700F, Jeol, Tokyo, Japan). The Fourier-transformed infrared (FTIR) spectra were obtained using Vertex 70, Bruker spectrometer. Raman measurement was carried out under 532 nm excitation using a Raman spectrometer (LabRam HR, Horiba, Tokyo, Japan) with a grating of 600 grooves/mm.

## 3. Result and Discussion

### 3.1. XRD Analysis

**Figure 1:**
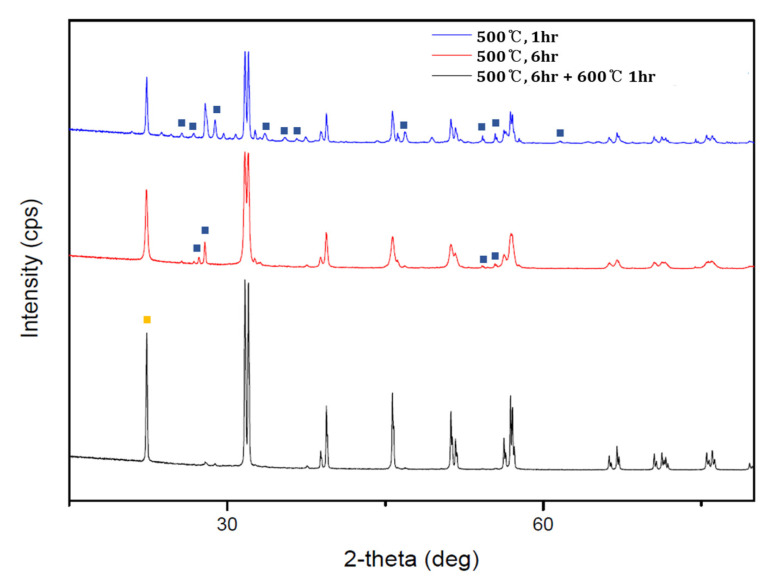
XRD spectra of the BiFeO_3_ samples heat-treated at different temperatures. Solid blue square represents Bi_2_O_3_ impurity phase and solid orange square represents pure BiFeO_3_ phase.

Typical XRD patterns of the BiFeO_3_ obtained using tartaric acid before and after its purification are shown in Figure 1. The phase belongs to rhombohedral BiFeO_3_, and the main byproducts are Bi_2_O_3_. As shown in the spectra, the intensity of the diffraction peaks increases with the increasing sintering temperature. XRD analysis shows that sintering temperature is an important factor in the synthesis of BiFeO_3_. Subsequent samples were manufactured to have high purity through the heat treatment of 500 °C for 6 h followed by 600 °C for 1 h sintering step. Hence, an optimized heat treatment condition of 500 °C for 6 h followed by 600 °C for 1 h was used for the synthesis of all the high-purity BiFeO_3_ samples for the fabrication of the DSSC.

### 3.2. BiFeO_3_ Hysteresis and SEM Analysis

**Figure 2:**
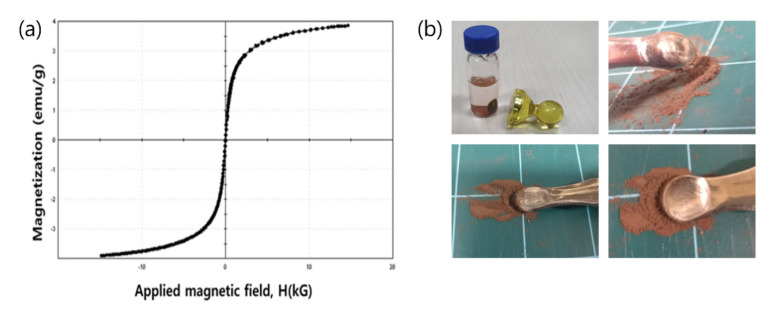
(**a**) Magnetic hysteresis loop of BiFeO_3_. (**b**) Optical photograph of magnetic properties of the BiFeO_3_.

BiFeO_3_ follows anti-ferromagnetic G-type ordering (spin of Fe^3+^ is antiparallel to that of the six neighboring Fe^3+^ ions), but G-type ordering is modified by cycloidal spiral modulation (λ = 62 nm) in the long region [19]. The hysteresis curve is obtained, as shown in Figure 2a. The magnetization M_s_ is 3.8 emu/g and the coercive field H_c_ represents a value of 8 Oe. The shape of the hysteresis curve confirms that the weakly ferromagnetic perovskite BiFeO_3_ sample has been obtained. Moreover, the weak ferromagnetic phenomena in the BiFeO_3_ sample can be observed, as shown in Figure 2b. This is the size-effect of BiFeO_3_ particles as a result of the increase in the area to volume ratio as the particle size decreases and the uncompensated spin of the surface increases the total magnetic moment [20,21,22,23].

**Figure 3:**
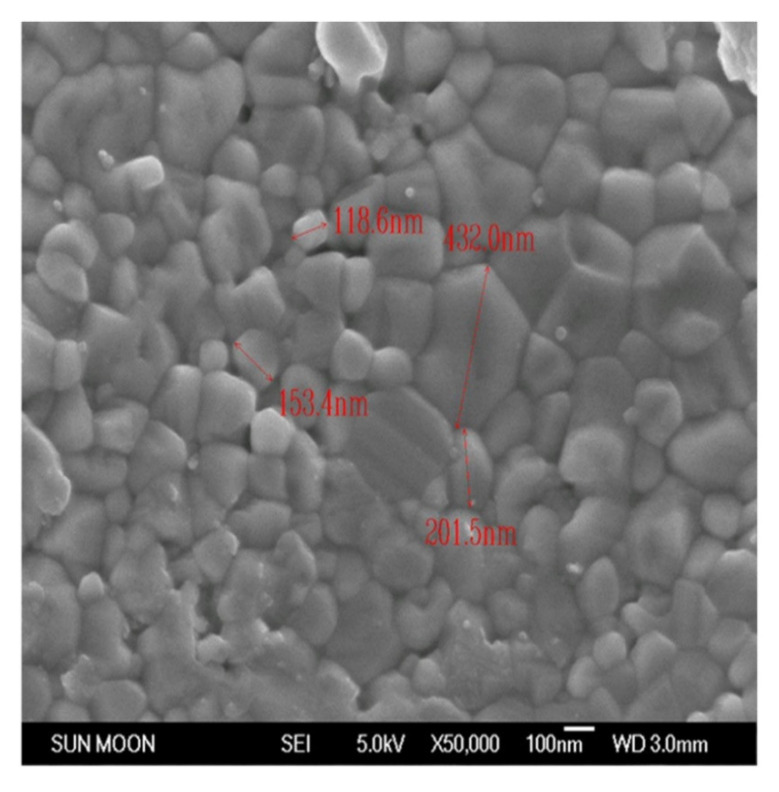
FESEM image of BiFeO_3_.

The particle morphology of the BiFeO_3_ sample, as shown in the FESEM image in Figure 3, also confirms that the particle size is in the range of 100–400 nm. Our observation confirms that the fabrication of high purity ferromagnetic BiFeO_3_ is possible even at room temperature.

### 3.3. Absorption Spectra

**Figure 4:**
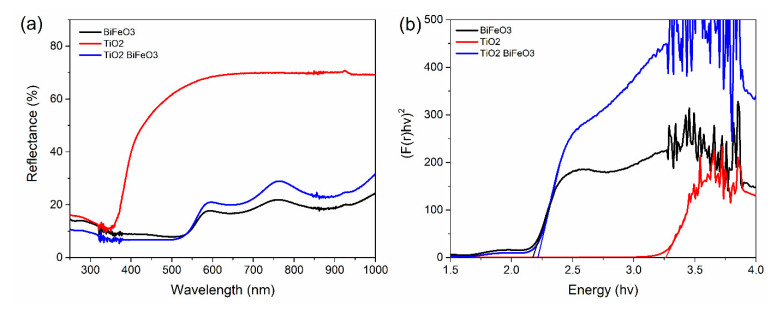
Optical properties of BiFeO_3_ (**a**) absorption spectrum, (**b**) Kubelka–Munk function for bandgap calculation.

The UV-visible absorption spectrum of the pure BiFeO_3_, TiO_2_, and TiO_2_-BiFeO_3_ samples are presented in Figure 4. It is evident from the absorption spectrum that BiFeO_3_ is a mostly visible light photocatalyst that can absorb energy in the visible spectral range from 400 nm to 600 nm. The bandgap was analyzed using Kubelka–Munk’s formula and Tauc plot. The Kubelka–Munk equation is

$$F\left(R\right)=\frac{K}{S}=\frac{{\left(1-R\right)}^{2}}{2R}$$

where *F*(*R*) is known as the Kubelka–Munk function and is given by *F*(*R*) = *K*/*S*, where *K* is the molar absorption coefficient *K* = (1 − *R*)^2^, *S* is the scattering factor *S* = 2*R*, and *R* being the reflectance of material equal to *R*. The optical bandgap energy *E_g_* is found by using the following relation:

*F*(*R*)*hν* = *A*(*hν* − *E_g_*)*^n^*

where *F*(*R*) is proportional to the absorption coefficient α, *A* is the constant, *h* is Planck’s constant, *ν* is the frequency of the incident light, and *n* is the parameter that depends on the transition characteristics of the semiconductor. That is *n* = 1/2 is for direct transition and *n* = 2 is for an indirect transition. The *E_g_* has been obtained by Tauc plot, as shown in Figure 4b of the extrapolation of the linear region of the spectra to α = 0 of a plot of (*F*(*R*)*hν*)^2^ versus *hν* [24]. The band gap of the TiO_2_-BiFeO_3_ sample was found to be 2.21 eV, which is much less than that of the pure TiO_2_ sample (3.26 eV). This value of the band gap of the TiO_2_-BiFeO_3_ sample is a suitable bandgap for manufacturing magnetized solar cells.

### 3.4. FTIR Spectrum

**Figure 5:**
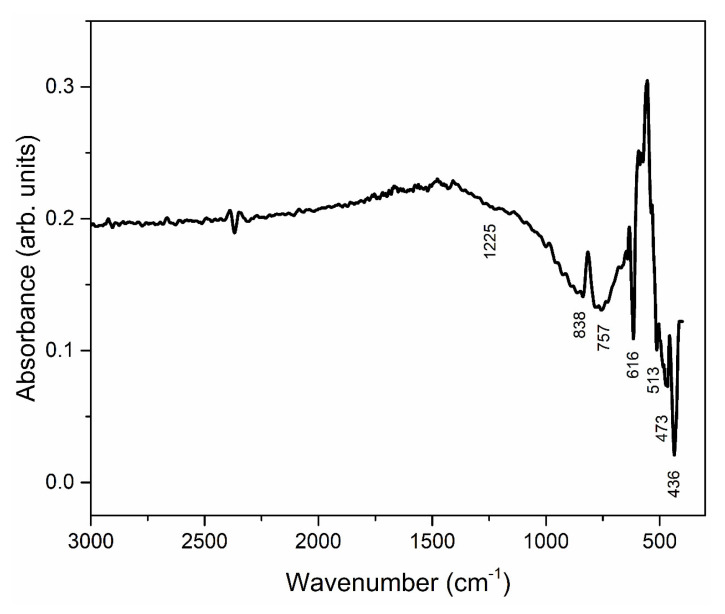
FTIR spectrum of BiFeO_3_.

The FTIR spectrum of the synthesized BiFeO_3_ sample is shown in Figure 5. Strong absorption peaks in the low-frequency range of 400–600 cm^−1^ are attributed to the Fe-O stretching and bending vibrations of the octahedral FeO_6_ groups indicating the formation of the BiFeO_3_ phase [25]. The presence of Fe-O vibrations in 400–600 cm^−1^ also confirms the existence of the perovskite structure of the BiFeO_3_. Another Fe-O absorption peak centered at 757 and 838 cm^−1^ suggests the high crystallinity of the BiFeO_3_ phase. The frequency band around 1225 cm^−1^ is probably due to the C-O stretching vibration appearing from organic molecules used for the synthesis of the BiFeO_3_. Weak absorption bands around 1550–1650 cm^−1^ correspond to the bending vibrations of H_2_O. The broadband below 3500 cm^−1^ is due to antisymmetric and symmetric stretching of H_2_O and O-H bond groups attached to the surface during measurement [26].

### 3.5. Raman Spectrum

**Figure 6:**
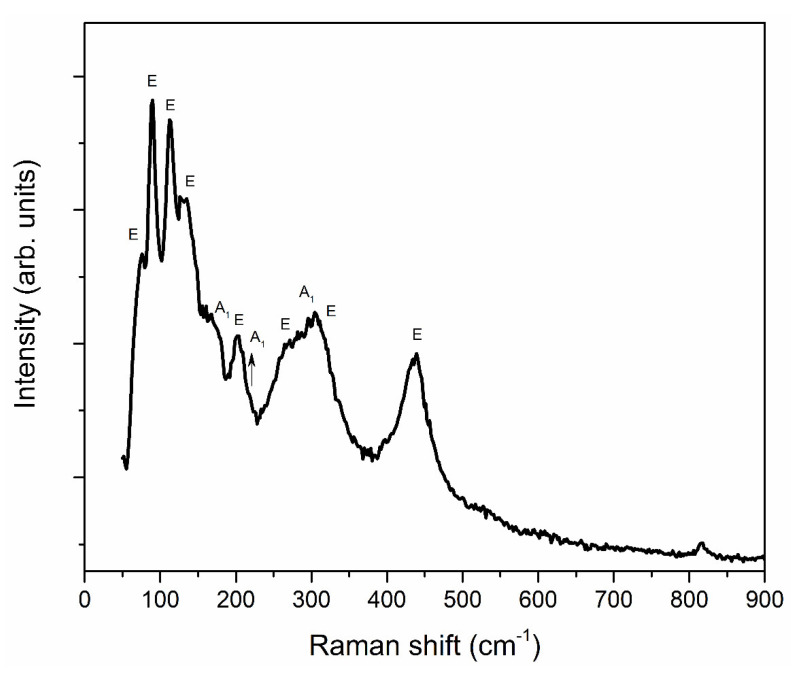
Raman spectrum of BiFeO_3_ powder ceramics.

The Raman spectrum of the BiFeO_3_ sample is shown in Figure 6. Raman spectrum of perovskite BiFeO_3_ consist of E and A_1_ Raman active phonon modes. As can be seen in the Raman spectrum, the first-order phonons are mostly confined to below 600 cm^−1^ with the strongest peaks around 89.4 and 112.5 cm^−1^. Another peak appears at an even lower frequency of 76.0 cm^−1^. The other Raman modes are observed at 134.6, 148.5, 155.5, 176.0, 219.0, 201.8, 265.2, 304.9, and 439 cm^−1^. The low-frequency vibration modes at 76 (E), 134.6 (E), and 176.0 (A_1_) are attributed predominantly to Bi motions. The mode at 219.0 (A_1_) corresponds to the atomic vibration of the O atom. The A_1_ modes corresponding to the vibration of Bi and O are related to the structural distortions away from the ideal cubic structure of BiFeO_3_. The modes are in agreement with the reported literature [26,27,28,29].

### 3.6. Photoelectrical Performance of an As-Fabricated and Magnetized TiO_2_-BiFeO_3_ DSSC

#### 3.6.1. As-Fabricated TiO_2_-BiFeO_3_ DSSC

The as-fabricated DSSC was analyzed using a solar simulator and an electrochemical analyzer, and the results for short-circuit current density (J_sc_), open-circuit voltage (V_oc_), fill factor (FF), and efficiency (η) are presented in Table 1. In the case of DSSC manufacturing, it is a continuous process that takes 3~4 days and the control of the variables occurring between them is very important. For example, TiO_2_ thickness, paste state, dye loading time, dye concentration, firing temperature, DSSC assembly, etc., were taken into account. The tabulated results were produced through the iterative experiments and optimization of the DSSC. Five TiO_2_-only DSSCs (TiO_2__1, TiO_2__2, TiO_2__3, TiO_2__4, and TiO_2__5) were prepared under identical conditions.

**Figure 7:**
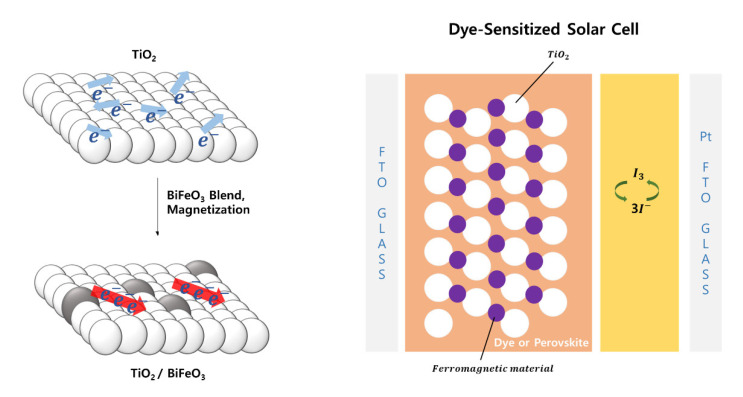
Schematic representation of control of the movement of electrons using a magnetic field.

The fabricated DSSCs have an average efficiency of 4%. In addition, the current density is 9.6 A/m^2^, V_oc_ is 0.653, and FF is 0.612. In general, the DSSC using TiO_2_ paste has an efficiency of between 3~5%. Therefore, it is considered that an appropriate DSSC has been fabricated. The results show that the current density and efficiency improved according to the magnetization direction. The mechanism of the improvement of the current density and efficiency are represented in Figure 7. Similarly, the BiFeO_3_ DSSC was manufactured to minimize the error and the results were compared.

#### 3.6.2. Magnetized TiO_2_-BiFeO_3_ DSSC

**Figure 8:**
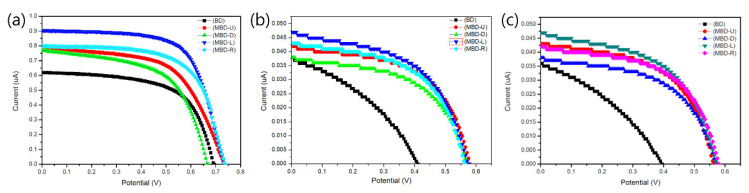
1% (**a**), 3% (**b**), and 5% (**c**) BiFeO_3_-TiO_2_ DSSCs I-V Curves.

**Figure 9:**
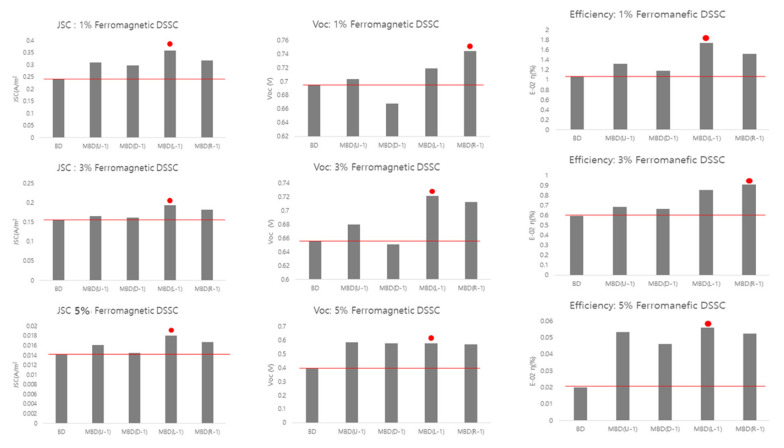
Ferromagnetic DSSC: J_sc_, V_oc_, and η Efficiency Comparison.

The J_sc_, V_oc_, FF, and η of the magnetized TiO_2_-BiFeO_3_ DSSC were measured using a solar simulator and an electrochemical analyzer. The results are shown in Figure 8 and Table 2. In the case of the magnetized TiO_2_-BiFeO_3_ DSSC, there are various variables affecting the efficiency, so the final result was calculated by taking the average value of at least five sample measurements (Figure 9). It can be seen that when the BiFeO_3_ content increases in the TiO_2_-BiFeO_3_ DSSC, the overall electrical properties decrease. It is difficult to make uniform paste due to the difference in particle sizes of TiO_2_ (~25 nm) and BiFeO_3_ (~100–400 nm) as well as the density difference of TiO_2_ and BiFeO_3_ during paste preparation. Therefore, it is possible that the decrease in efficiency is due to the adsorption in cracks and uneven, thin film formation during sintering. Moreover, it appears that the reason for the lower efficiency is also due to the BiFeO_3_ which acts as an impurity. However, after the magnetization, we observed the magnetization directional dependence of the efficiency due to the presence of the ferromagnetic BiFeO_3_, which helps to demonstrate the proposed concept of this study. Hence, as a general trend, it was confirmed that the samples magnetized in the left and right directions had relatively high electrical characteristics. This is because when the left and right magnetizations are perpendicular to the direction of the magnetic field and the current in the DSSC, the Hall effect is induced and the polarization occurs in the DSSC. As a result, the recombination rate decreases, and the electron mobility direction is limited, which suggests high potential and electrical properties (a higher V_oc_) [30,31,32,33]. In order to further support the result, the electrical impedance measurement of five samples in the first row of Table 2 (1%BiFeO_3_-TiO_2_ DSSC samples) with different magnetization directions was carried out and the result was consistent with that obtained from the I-V curve measurement. The result of the impedance measurement has been presented in Figure S5 (Supplementary Information). It can be seen that the impedance is in the increasing order for the magnetization direction as BD > MBD(D-1) > MBD(U-1) > MBD(R-1) > MBD(L-1) indicating the MBD(L-1) system has the maximum efficiency while the BD system has the least efficiency.

## 4. Conclusions

A ferromagnetic perovskite BiFeO_3_ incorporated TiO_2_ DSSC (TiO_2_-BiFeO_3_) was prepared and the effect of the concentration of BiFeO_3_ as well as the magnetization direction on the performance of the TiO_2_-BiFeO_3_ DSSC were investigated. The optimized TiO_2_-BiFeO_3_ DSSC has an average efficiency of 4%, J_sc_ of 9.6 A/m^2^, V_oc_ of 0.653, and FF of 0.612. As the BiFeO_3_ content increases in the DSSC, the overall electrical properties decrease, which may be due to the inhomogeneity caused by the particle size and density difference between TiO_2_ and BiFeO_3_ in the paste. It was found that the magnetic field induced the Hall effect which controls the direction of the electron movement. The enhancement of the efficiency of the TiO_2_-BiFeO_3_ DSSC is the evidence that two-dimensional material-like properties can be realized in the bulk of a TiO_2_-BiFeO_3_ DSSC (a 3-dimensional material).

## Figures and Tables

**Table 1:** TiO_2_ DSSC electrical characteristics data.

Sample	J_sc_ (A/m^2^)	V_oc_ (V)	FF	η (%)
TiO_2__1	10.7	0.662	0.610	4.31
TiO_2__2	9.35	0.642	0.607	3.64
TiO_2__3	10.1	0.650	0.600	4.94
TiO_2__4	9.25	0.651	0.624	3.76
TiO_2__5	8.63	0.657	0.619	3.51

**Table 2:** Sample efficiency comparison data before and after magnetization (U-up, D-down, L-left, R-right).

Sample		J_sc_ (A/m^2^) (±0.001)	V_oc_ (V) (±0.001)	FF (±0.001)	η (%)
**1%**	BD	0.242	0.695	0.633	1.07 × 10^−2^
	MBD(U-1)	0.309	0.713	0.586	1.32 × 10^−2^
	MBD(D-1)	0.297	0.668	0.594	1.18 × 10^−2^
	MBD(L-1)	0.358	0.719	0.676	1.74 × 10^−2^
	MBD(R-1)	0.317	0.744	0.644	1.52 × 10^−2^
**3%**	BD	0.156	0.656	0.583	0.60 × 10^−2^
	MBD(U-2)	0.165	0.680	0.61	0.68 × 10^−2^
	MBD(D-2)	0.162	0.651	0.627	0.66 × 10^−2^
	MBD(L-2)	0.194	0.722	0.611	0.85 × 10^−2^
	MBD(R-2)	0.182	0.713	0.703	0.91 × 10^−2^
**5%**	BD	0.014	0.397	0.35	0.02 × 10^−2^
	MBD(U-3)	0.016	0.585	0.564	0.05 × 10^−2^
	MBD(D-3)	0.015	0.576	0.552	0.05 × 10^−2^
	MBD(L-3)	0.019	0.577	0.540	0.06 × 10^−2^
	MBD(R-3)	0.017	0.568	0.554	0.05 × 10^−2^

## Data Availability

Not applicable.

## Supplementary Materials

The following supporting information can be downloaded at: https://www.mdpi.com/article/10.3390/ma15186367/s1.
