# Supplementary material for: Enhancement of Efficiency of a TiO2-BiFeO3 Dye-Synthesized Solar Cell through Magnetization
Source: Materials (Basel). 2022 Sep 13;15(18):6367. doi: 10.3390/ma15186367 (PMC9500914; doi:10.3390/ma15186367)
Supplement: Supplementary file 1 [file materials-15-06367-s001.zip › materials-1729392-supplementary.pdf]

Supplementary Information

Enhancement of Efficiency of a TiO_2-$BiFeO_3$ Dye-Synthesized Solar Cell Through Magnetization

Hyun Sik Kang[1], Woo Seoung Kim[1], Yuwaraj K. Kshetri[2], Hak Soo Kim[1,*], Hak Hee Kim[1,*]

[1]Department of Environmental and Bio-Chemical Engineering, Sun Moon University, Chungnam, 31460, Korea

[2]Research Center for Eco Multi-Functional Nano Materials, Sun Moon University, Chungnam, 31460, Korea

[*]Corresponding authors

E-mail: hskim@sunmoon.ac.kr (H.S.K.), hhkim@sunmoon.ac.kr (H.H.K.)

1. Synthesis of $BiFeO_3$

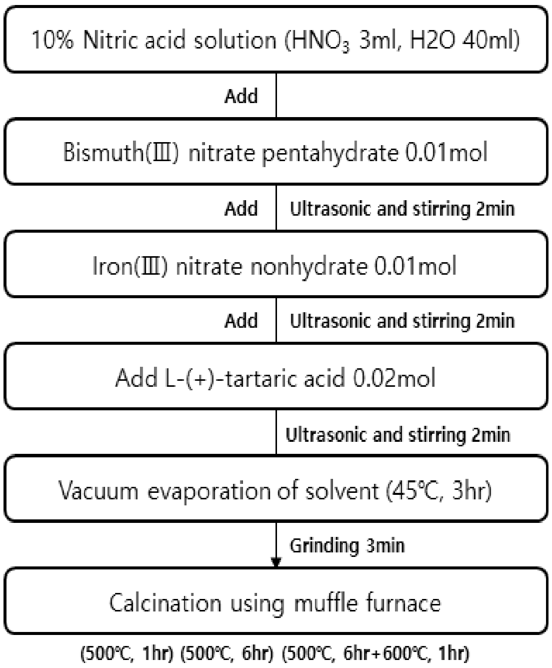

Figure S1. $BiFeO_3$ synthesis by sintering temperature and time (500℃ 1hr, 500℃ 6hr, 500℃ 6hr + 600 1hr)

2. Synthesis of TiO_2-$BiFeO_3$ paste

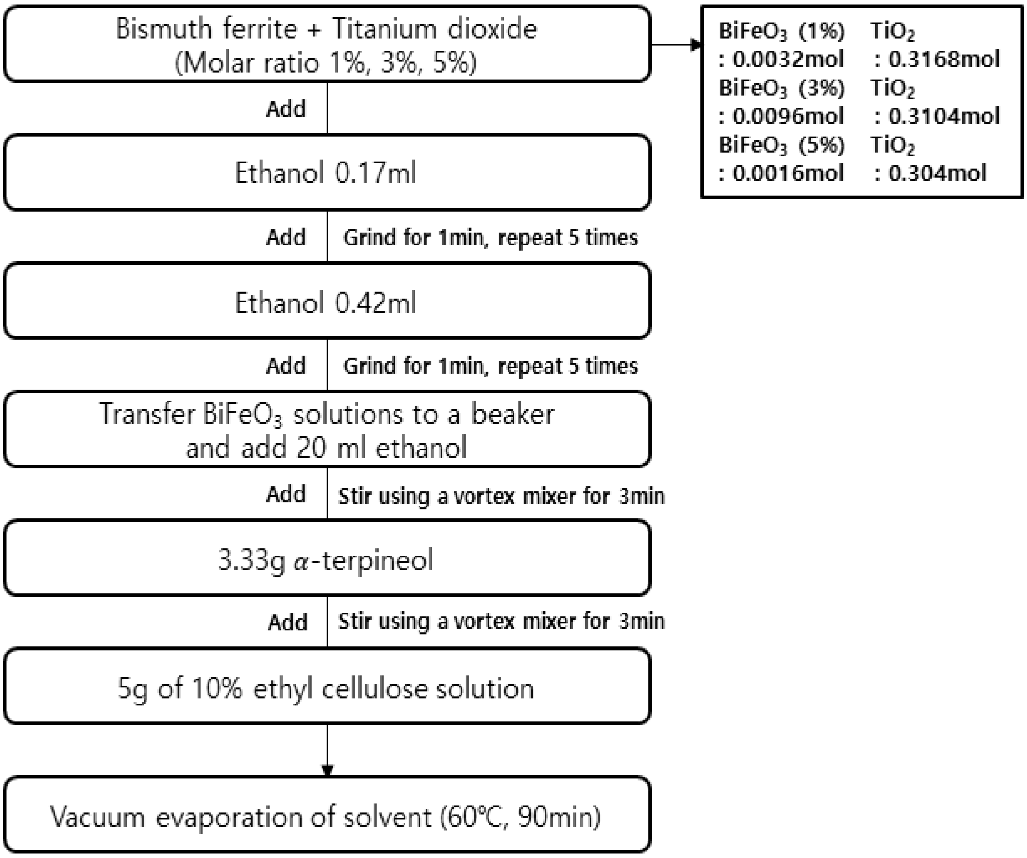

Figure S2. Experimental procedure of 1, 3 and 5% $BiFeO_3$-TiO_2 paste synthesis.

3. Fabrication of TiO_2-$BifeO_3$ DSSC

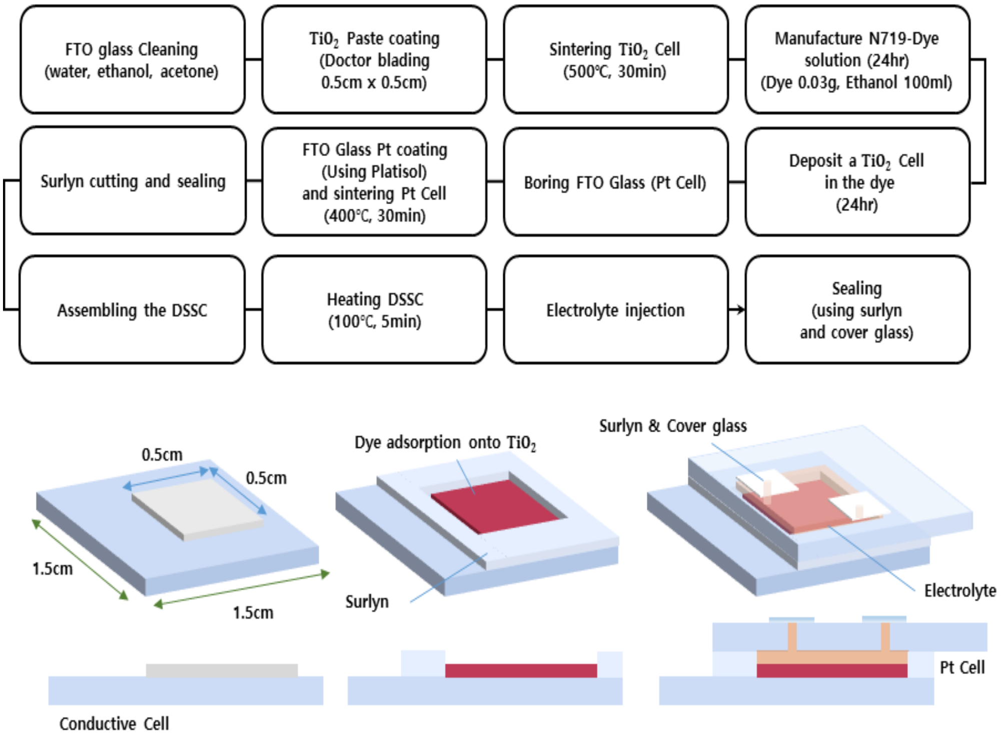

Figure S3. DSSC manufacturing method.

4. Fabrication of magnetized TiO_2-$BifeO_3$ DSSC

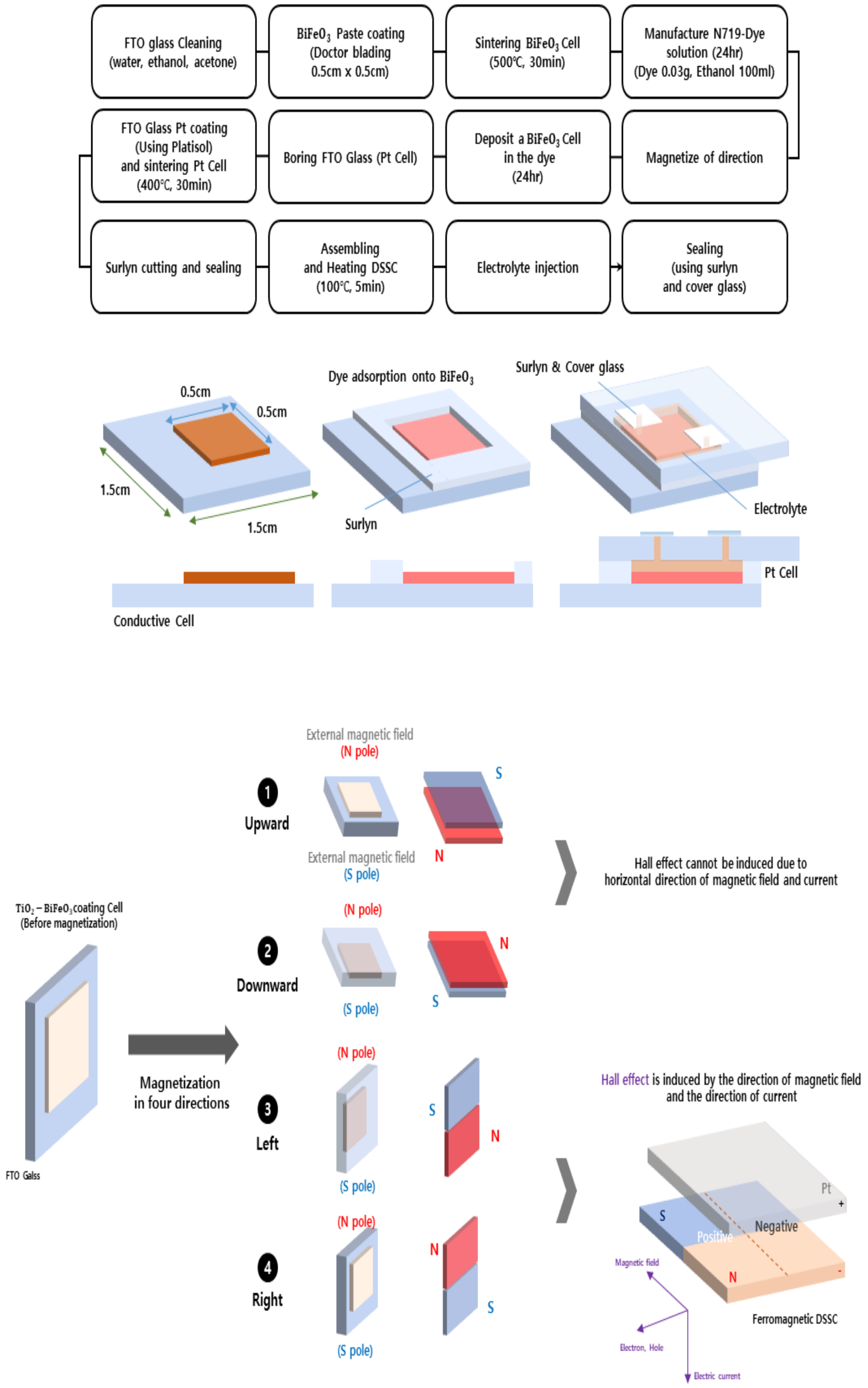

Figure S4. Magnetized TiO_2-$BifeO_3$ DSSC manufacturing method.

5. Electrical impedance measurement

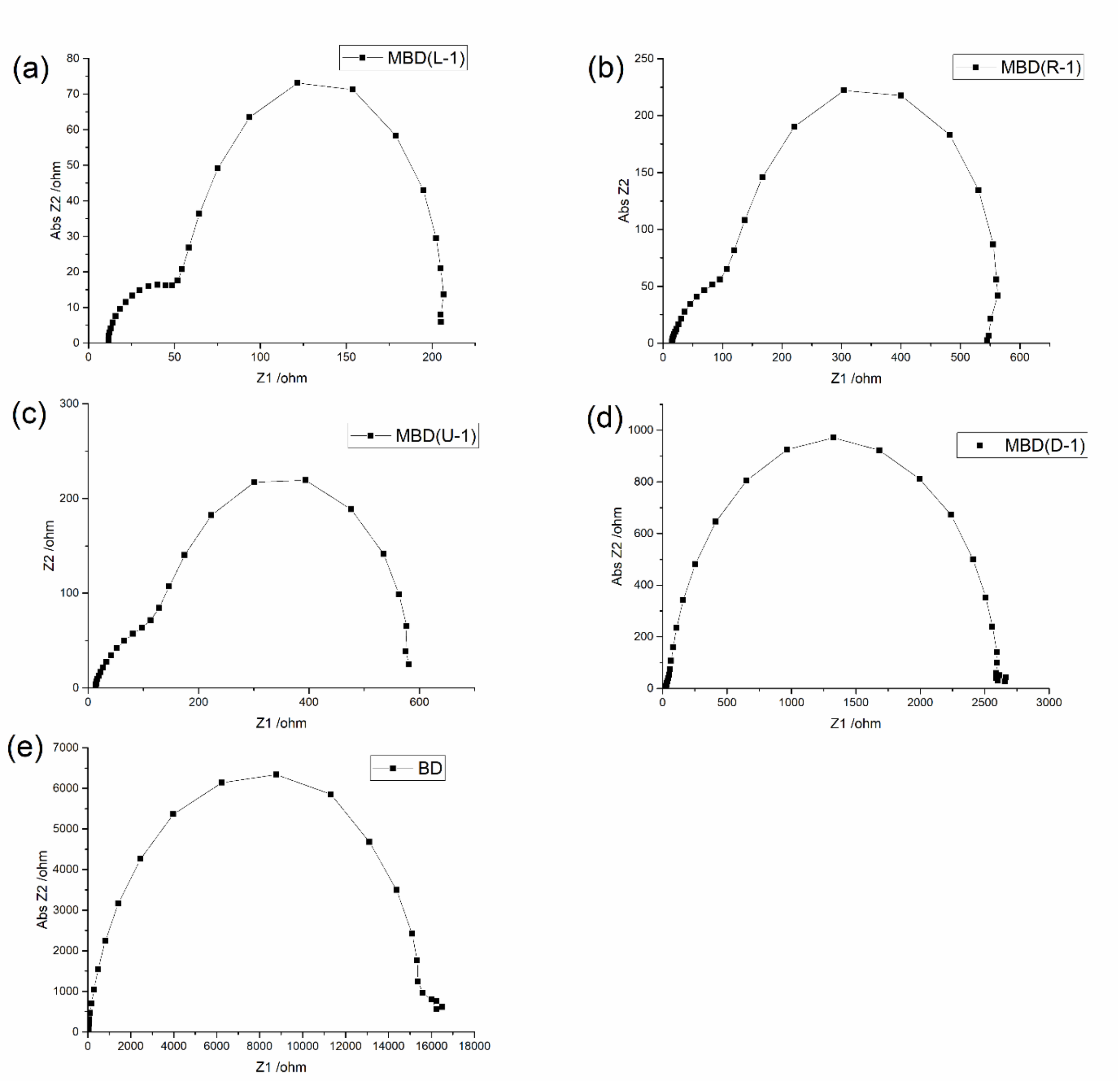

Figure S5. Impedance spectra of 1%$BiFeO_3$-TiO_2 DSSCs in different magnetization direction presented in Table 2 (a) MBD (L-1), (b) MBD (R-1), (c) MBD (U-1), (d) MBD (D-1), (e) BD.
